# Efficacy of Intravenous Nitrates for the Prevention of Coronary Artery Spasm During Pulsed Field Ablation of the Mitral Isthmus

**DOI:** 10.1161/CIRCEP.123.012426

**Published:** 2023-12-14

**Authors:** Roberto Menè, Serge Boveda, Domenico Giovanni Della Rocca, Vasileios Sousonis, Giampaolo Vetta, Sarah Zeriouh, Ioannis Doundoulakis, Andres Betancur, Mohamed Benadel, Nicolas Combes, Andrea Sarkozy, Gian Battista Chierchia, Carlo de Asmundis, Jean Paul Albenque, Stéphane Combes

**Affiliations:** Heart Rhythm Management Department, Clinique Pasteur, Toulouse, France (R.M., S.B., V.S., S.Z., A.B., M.B., N.C., J.P.A., S.C.).; Department of Medicine and Surgery, University of Milano-Bicocca, Milan, Italy (R.M.).; Heart Rhythm Management Centre, Postgraduate Program in Cardiac Electrophysiology and Pacing, Universitair Ziekenhuis Brussel-Vrije Universiteit Brussel, European Reference Network Guard-Heart, Brussels, Belgium (D.G.D.R., G.V., I.D., A.S., G.B.C., C.d.A.).

**Keywords:** atrial fibrillation, catheter ablation, coronary vasospasm, electroporation, isosorbide dinitrate, nitrates

Pulsed field ablation (PFA) is a promising strategy for the treatment of cardiac arrhythmias but carries a considerable risk of coronary artery spasm (CAS) when targeting anatomic structures adjacent to the coronary arteries, such as the mitral isthmus (MI) or the cavotricuspid isthmus (CTI).^1,2^ This complication could hinder the use of PFA for persistent atrial fibrillation or atrial flutters, where MI or CTI applications are often necessary. The aim of this study was to evaluate the efficacy of intravenous nitrates in preventing CAS during the PFA of the MI.

Starting from November 2022, we enrolled all patients undergoing PFA of the MI, as part of a previously described procedure for persistent atrial fibrillation,^3^ at 2 centers. Two minutes before the first application on the MI, 1 to 2 mg (depending on blood pressure) of isosorbide dinitrate (ISDN) was administered intravenously. The occurrence of procedural CAS was monitored and compared between this group (ISDN+ group) and a historical cohort of consecutive patients (November 2021 to November 2022) not pretreated with prophylactic nitrates (ISDN− group). CAS was defined as ST-segment changes during applications on the MI, resolving after nitrates administration or spontaneously within 5 minutes, in the absence of other causes of ischemia. All procedures were performed with the Farapulse system (5 biphasic pulses of 2.0 kV per application). The study was approved by the local institutional review boards, and patients provided written informed consent. Data were compared with Fisher exact, χ^2^, and *t* tests as appropriate (SPSS 29.0). *P*≤0.05 was considered significant. Data are available from the corresponding author upon reasonable request.

A total of 182 patients (age, 68±10 years; 29% females) were included. Baseline characteristics in both groups (ISDN+: n=79 and ISDN−: n=103) were similar, including the history of coronary artery disease (22.3% versus 13.9%; *P*=0.149) and angioplasty (12.6% versus 8.9%; *P*=0.421). Successful isolation of pulmonary veins and the posterior wall was achieved in 180 of 180 (100%) and 179 of 179 (100%) cases, respectively, while a bidirectional MI block was documented in 179 of 182 (98.3%) patients. More applications were delivered on the MI in the ISDN+ group (14 [interquartile range, 11–39] versus 12 [interquartile range, 8–28]; *P*=0.018).

All cases of CAS occurred in the ISDN− (10 of 103 patients, 9.7%) compared with none in the ISDN+ group (0 of 79 patients, 0%; *P*=0.005). Two patients developed significant hypotension, and 9 patients required administration of ISDN to resolve the ischemia. The first 2 cases of CAS were confirmed with coronary angiography (Figure). Patients developing CAS were more frequently hypertensive (100% versus 67%; *P*=0.013). Complications other than CAS were rare and were only observed in the ISDN− group before the first PFA application on the MI. These included coronary artery air embolism (n=1), cardiogenic shock (n=1), nonsustained ventricular tachycardia (n=1), and oxygen desaturation (n=1).

**Figure.**
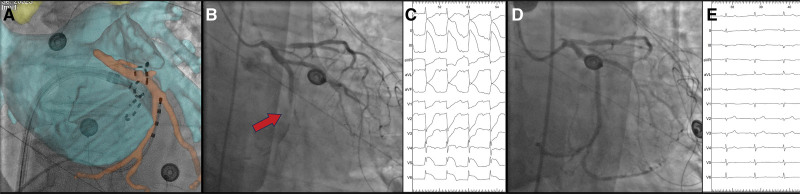
**Coronary artery spasm occurring during pulsed field ablation of the mitral isthmus. A**, Real-time integration of fluoroscopy with a computed tomography reconstruction of the left atrium (blue) and the left circumflex coronary artery (orange) in the left anterior oblique view showing the position of the Farawave catheter during the applications on the mitral isthmus. **B**, Coronary angiography demonstrating a spasm of the mid-left circumflex coronary artery (red arrow) following pulsed field ablation of the mitral isthmus. **C**, ECG during the coronary spasm event showing ST-segment elevation in the inferior and lateral leads. **D**, Coronary angiography showing spasm resolution following intracoronary administration of nitrates. **E**, ECG demonstrating complete regression of the ischemic signs following spasm resolution.

In the same period, 12 patients (age, 66±6 years; 17% females) underwent PFA of the CTI. Of these, 2 of 4 nonpretreated patients developed CAS compared with 0 of 8 pretreated patients (*P*=0.091). One CAS event resulted in ventricular fibrillation.^2^ Of note, no drug-related hypotension or allergic reactions following ISDN administration were seen in the total 87 (MI+CTI groups) pretreated patients.

We presented the largest published cohort of CAS following PFA and the first report on the efficacy of prophylactic ISDN in preventing this complication. Our study has 2 main findings. First, our data suggest that CAS occurs at a nonnegligible rate during the PFA of the MI. Second, in our cohort, prophylactic ISDN administration was safe and effectively reduced the risk of this complication.

Reddy et al^4^ previously reported a high rate of angiographically evident (100%) and clinically evident (20%) CAS in 5 nonpretreated patients who underwent systematic coronary angiography during PFA of the CTI. In the same study, prophylactic nitrates were highly effective in preventing CAS, limiting its occurrence to 3 of 15 patients pretreated with nitroglycerin. Notably, these CAS cases occurred during the last PFA applications, consistent with the diminishing effect of nitroglycerin (half-life, 1–2 minutes).

Here, we reported a comparable rate of clinically evident CAS during PFA of the MI among nonpretreated patients. Importantly, pretreatment with ISDN considerably decreased the risk of clinically evident CAS. As MI ablation procedures can be lengthy, we used the longer acting ISDN (half-life, ≈40 minutes).^5^ Additionally, more applications were delivered on the MI in the ISDN+ group, potentially reflecting greater operator confidence in ablating under the sustained effect of a longer lasting agent.

A couple of limitations should be acknowledged. First, this is a nonrandomized study; nevertheless, the consecutive enrollment of patients and the comparable baseline characteristics of the 2 groups mitigate the risk of biases. Second, a systematic angiographic evaluation was not performed, as our aim was to only focus on clinically significant CAS.

In conclusion, prophylactic ISDN appeared to be safe and effective in reducing the risk of CAS following the PFA of the MI. Future studies are needed to validate these results and to assess the efficacy of this approach in CTI ablation. In the meantime, we believe that prophylactic ISDN administration may be considered as a routine preventive strategy in all cases of PFA of the MI.

## ARTICLE INFORMATION

### Sources of Funding

None.

### Disclosures

Dr Boveda consults for Medtronic, Boston Scientific, MicroPort, and ZOLL. Dr Sarkozy is a consultant for Biosense Webster, Medtronic, Biotronik, and MicroPort. Dr Battista Chierchia received compensation for teaching purposes and proctoring from Medtronic, Abbott, Biotronik, Boston Scientific, and Acutus Medical. Dr de Asmundis receives research grants on behalf of the center from Biotronik, Medtronic, Abbott, LivaNova, Boston Scientific, AtriCure, Philips, and Acutus Medical and compensation for teaching purposes and proctoring from Medtronic, Abbott, Biotronik, LivaNova, Boston Scientific, AtriCure, Acutus Medical, and Daiichi Sankyo. Dr Albenque consults for Abbott and Volta Medical. The other authors report no conflicts.
